# Protective Effects of Quercetin and Eugenol on Auditory Dysfunction Associated With Oxidative Stress in a Type 2 Diabetic Rat Model

**DOI:** 10.1002/jbt.71118

**Published:** 2026-11-15

**Authors:** Aycan Baş, Cem Doğan, Işıl Albenİz

**Affiliations:** ^1^ Department of Biophysics, Faculty of Medicine Afyonkarahisar Health Sciences University Afyonkarahisar Türkiye; ^2^ Department of Biophysics, İstanbul Faculty of Medicine İstanbul University İstanbul Türkiye; ^3^ Department of Otorhinolaryngology–Head and Neck Surgery The Pearl International Hospital Doha Qatar

**Keywords:** auditory brainstem responses, auditory dysfunction, diabetes mellitus, eugenol, oxidative stress, quercetin

## Abstract

To assess oxidative stress–associated auditory dysfunction in a streptozotocin–nicotinamide (STZ–NA)‐induced type 2 diabetes mellitus (T2DM) rat model and to determine the protective effects of the phenolic compounds eugenol and quercetin. T2DM was induced in Sprague–Dawley rats using STZ–NA. Beginning 21 days after induction, eugenol or quercetin was administered for 28 consecutive days. Oxidative stress was assessed by measuring serum malondialdehyde (MDA) and superoxide dismutase (SOD) levels using ELISA. Auditory function was evaluated using auditory brainstem responses (ABR), elicited by click and tone‐burst stimuli at 8, 16, 24, and 32 kHz. ABR thresholds were elevated in T2DM animals across all frequencies, with the greatest impairment observed at 24–32 kHz. Serum MDA levels increased and SOD levels decreased in T2DM, whereas both interventions partially restored redox balance (*p* < 0.05). At 32 kHz, ABR thresholds correlated positively with MDA and negatively with SOD, with MDA identified as an independent predictor. The STZ–NA‐induced T2DM rat model exhibits frequency‐dependent auditory impairment and delayed neural conduction associated with oxidative stress. Eugenol and quercetin confer functional otoprotective effects, and high‐frequency ABR measurements may serve as indicators for early detection and monitoring of diabetes‐related auditory dysfunction.

## Graphical abstract

Hyperglycemia‐induced oxidative stress contributes to auditory dysfunction in experimental T2DM by increasing lipid peroxidation and weakening antioxidant defenses. Quercetin and eugenol attenuate oxidative stress, restore redox homeostasis, and improve auditory function, highlighting antioxidant therapy as a potential strategy against diabetes‐associated hearing impairment.

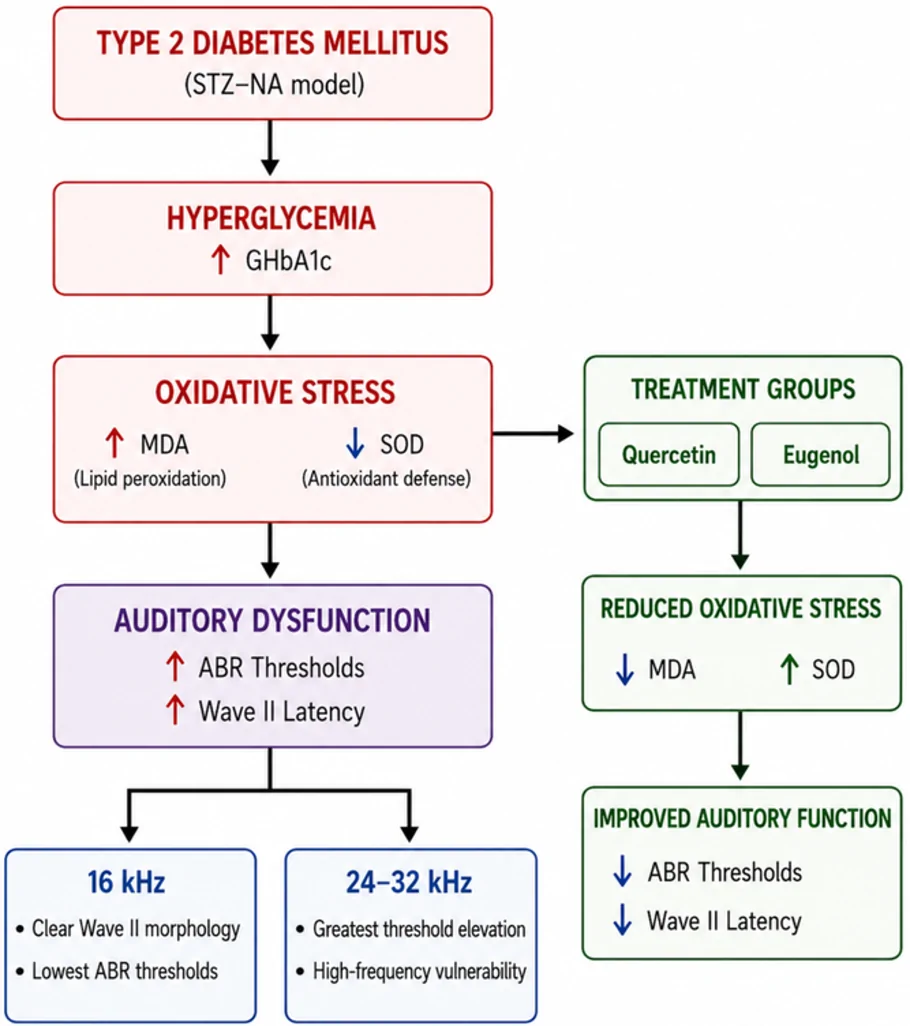

## 1 Introduction

Diabetes mellitus (DM) is a chronic and highly prevalent metabolic disorder characterized by persistent hyperglycemia resulting from impaired insulin secretion, insulin action, or both, with multisystem involvement [1, 2]. Approximately 90%–95% of individuals with DM have type 2 diabetes mellitus (T2DM), which develops primarily from insulin resistance accompanied by progressive *β*‐cell dysfunction [1]. DM is a major contributor to morbidity and mortality through both macrovascular complications, such as atherosclerosis and thrombosis, and microvascular complications, including nephropathy, retinopathy, and neuropathy [3].

Although optic and peripheral neuropathies are well‐established complications of DM, the association between DM and hearing loss remains unclear [4]. Increasing evidence, however, indicates a strong association between DM and sensorineural hearing loss (SNHL) [2]. Structural alterations, including thickening of the basement membrane in the stria vascularis, neuropathic changes, and vascular and neural injury within the inner ear, have been associated with diabetes‐related hearing impairment [5, 6]. Despite potential confounding from self‐reporting bias and environmental, demographic, and metabolic factors, the prevalence of hearing loss has been reported to be approximately twice as high in individuals with DM and 30% higher in individuals with prediabetes [7]. In T2DM, hyperglycemia accelerates auditory dysfunction through mechanisms involving microangiopathy, oxidative stress, and neural injury, causing structural deformities in the stria vascularis, spiral ganglion, and auditory hair cells [2, 8]. Histopathological studies have demonstrated capillary basement membrane thickening in the stria vascularis, spiral ganglion atrophy, and demyelination of the vestibulocochlear nerve as key contributors to diabetes‐related hearing loss [5, 9]. Furthermore, meta‐analyses and cohort studies have consistently shown associations among DM, insulin resistance, and hearing loss, with microangiopathy, mitochondrial dysfunction, and synaptopathy proposed as underlying mechanisms [5, 7, 9]. Collectively, these findings indicate that DM is a significant risk factor for hearing loss independent of age‐related changes and that heightened inflammatory activity and oxidative stress driven by reactive oxygen species (ROS) play central roles in diabetes‐associated auditory dysfunction.

Diabetic auditory dysfunction, a common microvascular complication of DM, is strongly influenced by oxidative stress and inflammation [10]. Accordingly, ROS scavengers and anti‐inflammatory agents have been proposed as potential strategies for preventing diabetes‐related auditory impairment. Polyphenols, which have potent antioxidant and anti‐inflammatory properties, have shown therapeutic efficacy against T2DM‐associated pathologies, including neuropathy, retinopathy, and cardiovascular complications, through insulin‐dependent mechanisms in both in vitro and in vivo studies [3, 10]. In diabetic neuropathy, changes in glutamatergic signaling, accumulation of advanced glycation end products, polyol pathway activation, and ion channel dysfunction contribute substantially to the initiation and progression of neuronal damage due to oxidative stress. Disruption of endogenous antioxidant defense systems, including catalase (CAT), superoxide dismutase (SOD), and glutathione (GSH), exacerbates cellular injury. At the same time, excessive ROS accumulation promotes protein and lipid damage and compromises myelin stability [10]. Consequently, SOD activity and malondialdehyde (MDA), a lipid peroxidation byproduct, are widely used biomarkers in studies of auditory dysfunction [11]. Additionally, polyphenolic compounds have been shown to modulate cochlear blood flow, attenuate oxidative damage and inflammation, and exert protective effects on the auditory system [3].

Phenolic compounds mitigate diabetes‐related complications by inhibiting enzymes involved in ROS production and exerting strong antioxidant and free radical–scavenging effects. Quercetin and eugenol, in particular, exhibit antioxidant and anti‐inflammatory activities, promote free radical scavenging, regulate mitochondrial apoptosis pathways, and protect against oxidative injury. Quercetin has been shown to protect auditory hair cells from gentamicin‐induced ototoxicity in rats [12] and to reduce noise‐induced hearing loss in guinea pigs [13]. In experimental diabetic models, eugenol has been reported to improve neural and vascular function through its antioxidant and anti‐inflammatory properties [14] and to attenuate oxidative stress by reducing lipid peroxidation and enhancing SOD and glutathione peroxidase (GPx) activities [15, 16]. Quercetin has also been shown to prevent diabetic complications by inhibiting lipid peroxidation, platelet aggregation, and vascular permeability and by modulating lipid metabolism and the expression of proinflammatory cytokines [17]. Furthermore, both quercetin and eugenol have demonstrated antioxidant, antidiabetic, and osteoprotective effects in an STZ–NA‐induced T2DM model, supporting their therapeutic potential in diabetes‐related tissue damage [18].

Auditory brainstem responses (ABR) are a well‐established electrophysiological method for assessing auditory dysfunction. During an ABR test, electrical activity generated along the auditory nerve and brainstem in response to click or tone‐burst stimuli delivered through headphones is recorded using surface electrodes placed on the scalp [11]. The resulting ABR waves comprise five positive peaks occurring within the first 10 ms after stimulus onset, reflecting neural conduction from the cochlea to the brainstem and corresponding to distinct anatomical generators. In rats, Wave II is the most prominent component and is commonly used as the primary reference for hearing threshold determination [11, 19]. The use of animal models that closely replicate human clinical conditions is essential for elucidating disease pathophysiology. Streptozotocin (STZ) combined with nicotinamide (NA) is a widely accepted experimental model for nonobese T2DM. STZ induces pancreatic *β*‐cell injury, whereas NA partially preserves *β*‐cell function by inhibiting poly(ADP‐ribose) polymerase‐1 (PARP‐1), thereby producing a metabolic phenotype that closely resembles human T2DM [20].

To the best of our knowledge, auditory function in an STZ–NA‐induced T2DM model has not previously been examined using a detailed frequency‐specific ABR protocol that incorporates Wave II latency analysis alongside systemic oxidative stress biomarkers, including MDA and SOD. Moreover, the comparative effects of quercetin and eugenol on auditory pathway function in this model have not been evaluated. Accordingly, this study integrates ABR measurements with redox biomarkers to characterize diabetes‐related auditory dysfunction and to investigate the potential functional protective and antioxidant effects of quercetin and eugenol.

## 2 Materials and Methods

### 2.1 Experimental Subjects

A total of 37 adult male Sprague–Dawley rats, aged 10–12 weeks and weighing 250–280 g, were obtained from the Aziz Sancar Institute of Experimental Medicine Research, Istanbul University. All animal care and experimental procedures were conducted at ASDETAE. Throughout the study, animals were housed under standard laboratory conditions (23°C ± 2°C, 55% ± 5% humidity, and a 12 h light/dark cycle) and fed standard rat chow and tap water ad libitum.

### 2.2 Ethical Approval

All animal care and handling procedures were approved by the Local Ethics Committee for Animal Experiments of Istanbul University (Approval No. 2016/26) and conducted in accordance with national regulations for the care and use of laboratory animals. The procedures were consistent with the principles of the European Directive 2010/63/EU and were reported in accordance with the ARRIVE guidelines.

### 2.3 Experimental Groups

Eligibility was determined by otoscopic examination and baseline ABR. Rats were included if they exhibited no cerumen obstruction in the external auditory canal, intact tympanic membranes, and detectable Wave II responses at 30 dB SPL using click stimuli. The animals were acclimatized to the experimental environment for 48 h and then randomly assigned to four groups.

Given the known mortality associated with chemically induced diabetes models and the potential for unsuccessful diabetes induction, each diabetic group was initially set to include 10 animals. During the experimental period, mortality among rats with experimentally induced T2DM ranged from 25% to 30%.

T2DM was induced in 30 rats by intraperitoneal (i.p.) administration of STZ (Sigma, S0130) and NA (Sigma, 72340). These animals were randomly allocated to three diabetic groups (*n* = 10 per group), and seven rats served as healthy controls. As illustrated in the experimental timeline (Figure 1), a 21‐day period was allowed for the development of diabetic neuropathy, after which daily oral gavage administration of either eugenol (Sigma, E51791) or quercetin (Sigma, Q4951) was continued for 28 consecutive days.

Group I: Healthy control group (*n* = 7).

Group II: T2DM group (*n* = 7).

Group III: T2DM + eugenol treatment group (*n* = 7).

Group IV: T2DM + quercetin treatment group (*n* = 7).

**Figure 1:**
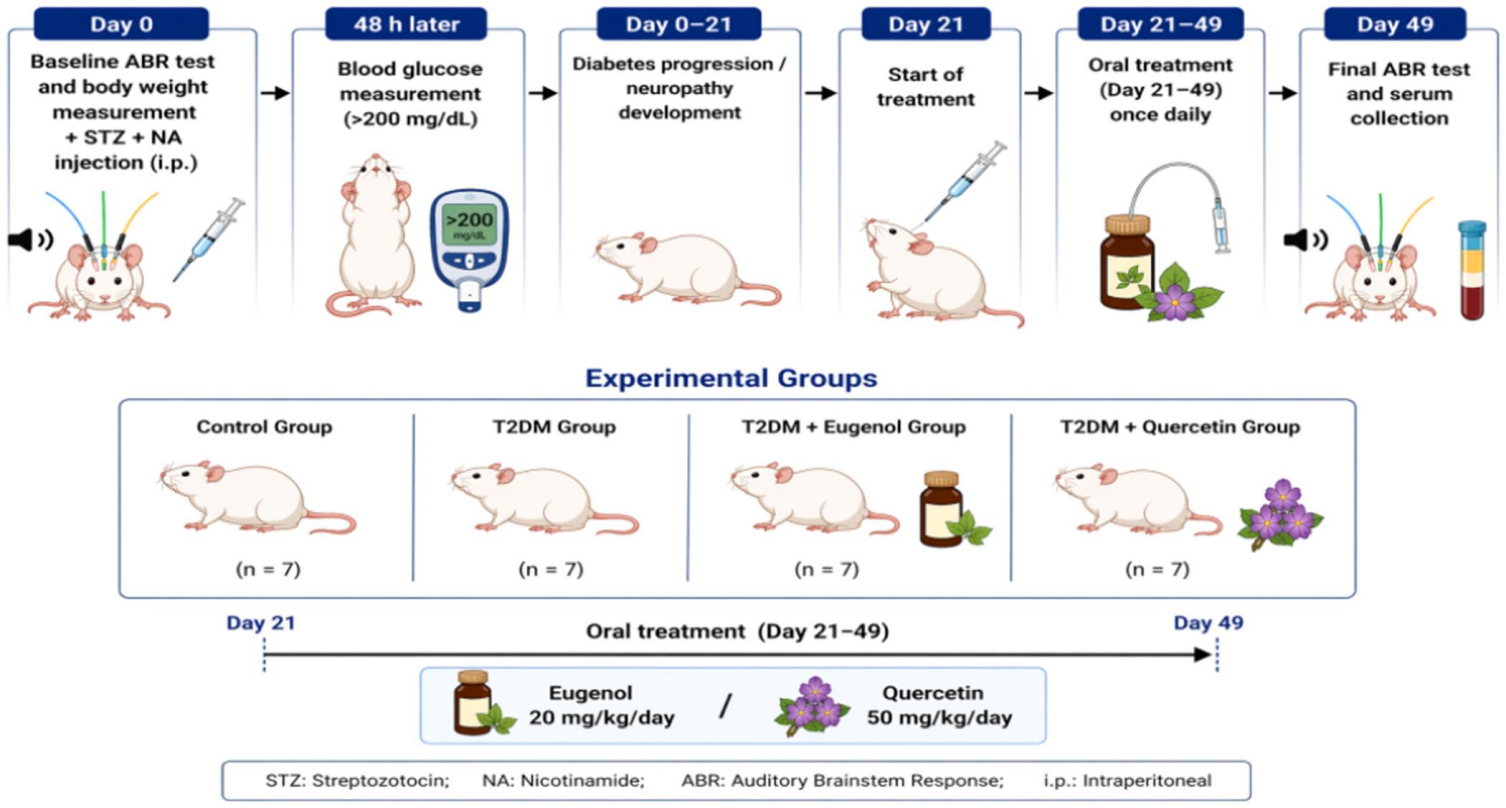
Schematic representation of the experimental design.

Timeline illustrating baseline ABR testing, STZ–NA administration for induction of T2DM, blood glucose verification, grouping of animals, initiation of oral antioxidant treatment (eugenol or quercetin) on Day 21, and final ABR testing with serum collection on Day 49.

### 2.4 Induction of Experimental T2DM

After a 12 h fasting period with free access to water, the rats received an i.p. injection of NA (120 mg/kg) freshly prepared in 0.2 mL of physiological saline. Twenty minutes later, STZ (65 mg/kg), freshly prepared in 0.2 mL of saline and protected from light, was administered intraperitoneally [18, 20] using a 26‐gauge insulin syringe. After injections, animals were allowed free access to food and water. The control group received an equivalent volume of physiological saline through the same route. Forty‐eight hours after injection, blood glucose levels were measured from the tail vein using a glucometer (Accu‐Chek Performa Nano). Rats with glucose levels exceeding 200 mg/dL were classified as diabetic and included in the study. Blood glucose levels and body weight were monitored weekly at consistent times (09:00–10:00) throughout the experimental period and recorded for subsequent statistical analysis.

### 2.5 Administration of Antioxidants

Antioxidant treatment commenced 21 days after T2DM induction and continued for 28 days. Group III received eugenol at 20 mg/kg, and Group IV received quercetin at 50 mg/kg, both administered once daily by oral gavage as previously reported [18]. Eugenol and quercetin were dissolved in a 3:1 saline–ethanol vehicle, and doses were adjusted weekly based on body weight. Groups I and II received 0.3 mL of the vehicle solution by gavage daily. All solutions were freshly prepared and administered at the same time each day (09:30–10:00) from Day 21 until the end of the experimental period.

### 2.6 Auditory Brainstem Response Testing

The ABR testing was conducted in a sound‐attenuated room with ambient noise levels not exceeding 45 dB SPL. Rats were anesthetized by i.p. injection of ketamine (50 mg/kg; Ketalar, Pfizer) and xylazine (5 mg/kg; Rompun, Bayer) before testing. During recording, body temperature was continuously monitored using a rectal probe and maintained at 37.5°C ± 1°C.

Auditory function was assessed using the SmartEP system (Intelligent Hearing Systems, USA). Animals were placed in the prone position, and recordings were obtained using high‐frequency transducer earphones and subdermal needle electrodes. Reference electrodes were positioned at each mastoid, and the ground electrode was positioned at the vertex. Electrode impedance was maintained below 5 kΩ.

As shown in Figure 2, ABR thresholds and waveform morphology were evaluated using tone‐burst stimuli at 8, 16, 24, and 32 kHz, starting at 80 dB SPL and decreasing in 5 dB increments until no reproducible response was detected. Thresholds were verified by two repeated measurements. Wave II latency was analyzed at 80 dB SPL for both tone‐burst and click stimuli across all frequencies in all groups [11, 19].

**Figure 2:**
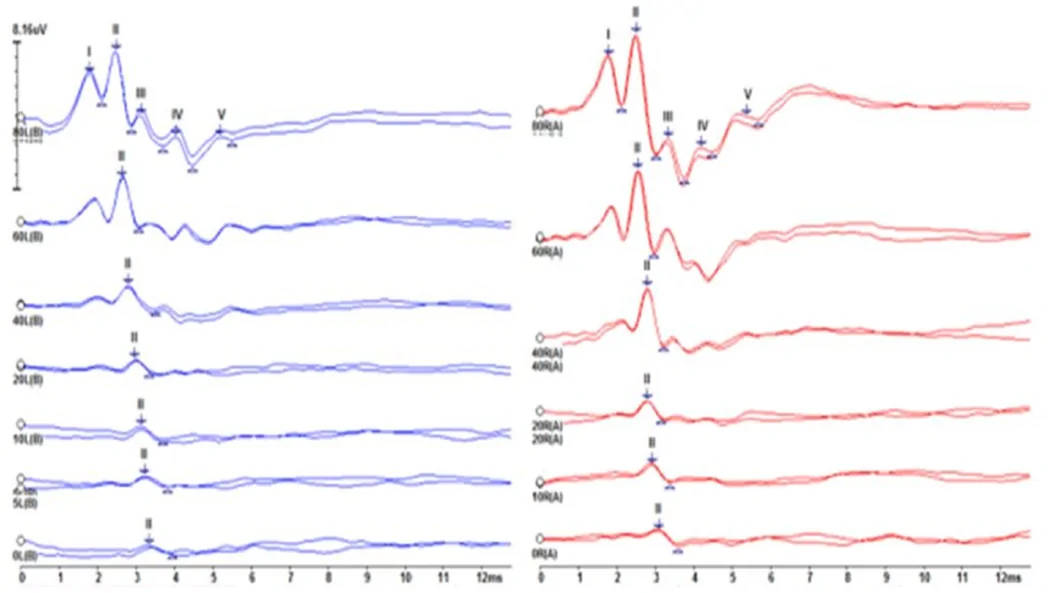
ABR test screen illustrating threshold determination and waveform morphology in response to 16 kHz stimulation in the right and left ears of the control group.

Each stimulus was presented 1024 times with alternating polarity, using repetition rates of 11.1/s for click stimuli and 21.1/s for tone‐burst stimuli. The analysis window was set to 0–12 ms. Signals were band‐pass filtered at 30 Hz–3 kHz, with a noise rejection level of ±25 µV and a sensitivity of 50 µV. Data were analyzed using SmartEP software (version 5.20). Recordings at 16 kHz are shown because of their superior waveform clarity. ABR testing was performed bilaterally, and the data were averaged per animal (*n* = 7 per group).

### 2.7 Blood Sample Collection

Immediately after ABR testing, approximately 5 mL of blood was collected by intracardiac puncture under anesthesia and allowed to clot at room temperature for 15–20 min. Samples were then centrifuged at 3000 rpm for 10 min, after which serum was separated and stored at −20°C until biochemical analysis.

### 2.8 Determination of GHbA1c, MDA, and SOD Levels by ELISA

Serum levels of glycated hemoglobin A1c (GHbA1c), MDA, and SOD were quantified using commercial ELISA kits from Shanghai Sunred Biological Technology Co. Ltd. (Cat. Nos. 201‐11‐0585, 201‐11‐0157, and 201‐11‐0169, respectively). Five serial dilutions of stock standards were prepared. For each assay, 50 µL of each standard solution and 40 µL of serum samples were added to 96‐well microplates. Antibody solution (10 µL) was added to all wells except blanks and standards, and 50 µL of Streptavidin‐HRP was added to all wells except blanks. Plates were sealed and incubated at 37°C for 60 min with gentle agitation.

The concentrated washing buffer (20 mL) was diluted 1:30 with distilled water. After incubation, wells were aspirated and washed five times with 0.35 mL of washing solution. Subsequently, 50 µL of chromogen A and 50 µL of chromogen B were added to each well, and plates were incubated in the dark at 37°C for 10 min. The reaction was stopped by adding 50 µL of stop solution, and absorbance was measured at 450 nm within 15 min using an ELISA plate reader. Analyte concentrations were calculated from standard curves.

### 2.9 Statistical Analysis

Data were analyzed using IBM SPSS Statistics for Windows, version 21.0. Continuous variables are presented as mean ± standard deviation (SD) for descriptive purposes. Normality and variance homogeneity were assessed using the Shapiro–Wilk and Levene's tests, respectively. Data demonstrating normal distribution and homogeneity of variances were analyzed using one‐way analysis of variance (ANOVA) followed by Tukey's post hoc test, whereas variables that did not meet parametric assumptions were analyzed using the Kruskal–Wallis test followed by Dunn's multiple comparisons test. Relationships between variables were evaluated using Spearman's rank correlation coefficient (*r*_s_). A multivariable linear regression analysis was performed to identify independent predictors, and model assumptions, including residual normality and multicollinearity, were verified. Statistical significance was set at *p* < 0.05.

### 2.10 AI Usage Declaration

During the preparation of this manuscript, the authors used OpenAI's ChatGPT, powered by GPT‐5, to improve the readability and English‐language quality of selected passages and to assist in creating the graphical abstract and the schematic representation of the experimental protocol presented in Figure 1, based on scientific content and detailed instructions provided by the authors. All AI‐assisted material was reviewed, verified, and revised by the authors. AI was not used for study design, data generation or modification, statistical analysis, interpretation of findings, or formulation of scientific conclusions.

## 3 Results

### 3.1 Metabolic Validation of the Experimental T2DM Model

Metabolic validation of the experimental T2DM model was confirmed by longitudinal measurements of body weight and fasting blood glucose levels (see Supporting Information S2: Tables 1 and 2).

### 3.2 GHbA1c, MDA, and SOD ELISA Test Results

Serum GHbA1c and MDA levels were significantly higher, whereas SOD levels were significantly lower in the T2DM group compared with the control group (*p* < 0.01). Eugenol treatment significantly reduced GHbA1c and MDA levels and increased SOD levels compared with untreated T2DM (*p* < 0.05), while quercetin significantly reduced MDA levels and increased SOD levels (*p* < 0.05) but did not significantly affect GHbA1c (*p* > 0.05). All pairwise comparisons were performed; nonsignificant differences are not indicated in the figure.

### 3.3 Auditory Function Assessment–ABR Test Results

To evaluate the effects of antioxidant treatment on auditory function following diabetes induction, hearing thresholds were assessed using ABR testing, a well‐established method for evaluating auditory function in rats.

ABR recordings were obtained using click and tone‐burst stimuli at 8, 16, 24, and 32 kHz, presented at 80 dB SPL. Across all experimental groups, robust Wave II responses were consistently identified bilaterally, as shown in representative recordings in Figure 3.

This figure provides a reference waveform for threshold determination and waveform morphology. Comparative representative waveforms from all experimental groups are provided in the Supporting Material (Supporting Information S1: Figures 1–4).

During ABR testing, tone‐burst stimuli at 8, 16, 24, and 32 kHz were presented to the control and experimental groups, starting at 80 dB SPL and decreasing in stepwise fashion until no response was detected. When responses were absent, stimulus intensity was increased in 5 dB increments until a clear, reproducible waveform was observed, and auditory thresholds were defined accordingly. Threshold values for the right and left ears at each frequency in the control and experimental groups are shown in Figure 4a–d, respectively.

As shown in Figure 4a–d, auditory thresholds were significantly elevated in the diabetic group compared with controls at 8, 16, 24, and 32 kHz (*p* < 0.001). At 8 kHz, the diabetic group exhibited significantly elevated ABR thresholds compared with controls (*p* < 0.001), while both eugenol and quercetin treatments significantly attenuated this increase relative to the diabetic group (*p* < 0.01). At 16 kHz, the quercetin‐treated group also demonstrated a modest but significant increase in thresholds compared with controls (*p* < 0.05). At 24 kHz, both the eugenol‐ and quercetin‐treated groups showed higher thresholds than the controls (*p* < 0.05). At 32 kHz, ABR thresholds were markedly elevated in the diabetic group compared with controls (*p* < 0.001). Both treatment groups showed significant reductions relative to the diabetic group, with a stronger improvement observed in the eugenol‐treated group (*p* < 0.01) and a moderate reduction in the quercetin‐treated group (*p* < 0.05), although thresholds remained higher than those of the control group (*p* < 0.001). All pairwise comparisons were performed; nonsignificant differences are not indicated in the figure.

At 32 kHz, ABR threshold values were positively correlated with serum MDA levels (Spearman *r*_s_ = 0.44, *p* = 0.020) and negatively correlated with serum SOD levels (*r*_s_ = −0.39, *p* = 0.041). In a multivariable linear regression model predicting ABR thresholds at 32 kHz, serum MDA was an independent positive predictor (*β* = 4.42 dB per unit increase; 95% CI: 0.05–8.79; *p* = 0.048), whereas SOD showed a negative trend that did not reach statistical significance (*β* = −0.89 dB; 95% CI: −1.89 to 0.11; *p* = 0.081). The overall model was significant (*R* ^2^ = 0.223; *p* = 0.018).

Table 1 presents the frequency‐specific auditory threshold values (dB SPL) obtained from the control, T2DM, eugenol, and quercetin groups in response to tone burst stimuli at 8, 16, 24, and 32 kHz.

**Table 1:** Frequency‐specific auditory threshold values (dB SPL) in the control and experimental groups at 8, 16, 24, and 32 kHz stimuli.

Frequency	Control	T2DM	Eugenol	Quercetin	*p*
8 kHz	18.57 ± 3.06	33.93 ± 17.00	22.50 ± 5.46	21.43 ± 4.97	< 0.001
16 kHz	0.71 ± 1.82	17.86 ± 16.02	11.43 ± 14.06	12.86 ± 13.26	< 0.001
24 kHz	26.43 ± 9.69	48.57 ± 14.47	38.21 ± 10.12	40.71 ± 12.54	< 0.001
32 kHz	28.21 ± 11.70	70.00 ± 14.68	51.79 ± 16.94	55.71 ± 17.08	< 0.001

*Note:* Values are expressed as mean ± SD. Group comparisons were performed using the Kruskal–Wallis test. A *p* < 0.05 was considered statistically significant.

As shown in Table 1 and Figure 5, auditory thresholds were significantly elevated in the T2DM group compared with controls across all tested frequencies (*p* < 0.001). The most pronounced increases occurred at higher frequencies (24 and 32 kHz). Notably, both eugenol‐ and quercetin‐treated groups exhibited significantly lower thresholds than the untreated T2DM group, approaching control values.

Table 2 presents the Wave II latency values obtained from the control, T2DM, eugenol, and quercetin groups in response to click and tone burst stimuli at 8, 16, 24, and 32 kHz (80 dB SPL).

**Table 2:** Wave II latency values (ms) in response to click and tone burst stimuli (8, 16, 24, and 32 kHz at 80 dB SPL) across control, T2DM, eugenol, and quercetin groups.

Stimulus type	Control	T2DM	Eugenol	Quercetin	*p*
Click	2.27 ± 0.12	2.39 ± 0.10	2.31 ± 0.06	2.32 ± 0.08	0.017*
8 kHz	2.60 ± 0.10	2.74 ± 0.20	2.69 ± 0.17	2.69 ± 0.11	0.149
16 kHz	2.48 ± 0.09	2.59 ± 0.10	2.51 ± 0.09	2.57 ± 0.10	0.018*
24 kHz	2.67 ± 0.22	2.76 ± 0.16	2.70 ± 0.16	2.73 ± 0.19	0.819
32 kHz	2.63 ± 0.19	2.77 ± 0.23	2.74 ± 0.22	2.75 ± 0.21	0.316

*Note:* Values are expressed as mean ± SD. Group comparisons were performed using one‐way ANOVA followed by Tukey's post hoc test.

* *p* < 0.05 was considered statistically significant.

Latency values in Table 2 and Figure 6 were consistently higher in the T2DM group compared with controls across all stimulus frequencies. Significant prolongation of Wave II latency was observed in response to click (*p* = 0.017) and 16 kHz (*p* = 0.018) stimuli, while no significant differences were found at 8, 24, or 32 kHz (*p* > 0.05). Eugenol‐ and quercetin‐treated groups showed intermediate latency values between the control and T2DM groups.

## 4 Discussion

Diabetes‐related auditory dysfunction arises from interacting microvascular and neurodegenerative processes driven primarily by chronic hyperglycemia and oxidative stress. Using an STZ–NA‐induced T2DM model that was metabolically validated by sustained hyperglycemia and characteristic changes in body weight, this study provides the first comparative evaluation of quercetin and eugenol in attenuating diabetes‐associated auditory impairment.

The cochlea is particularly susceptible to oxidative injury because of its high metabolic demand and limited vascular supply. Reduced antioxidant capacity and mitochondrial dysfunction promote a self‐perpetuating cycle of energy depletion, resulting in excessive ROS production, cellular damage, and apoptosis [21, 22]. Oxidative stress, inflammation, calcium dysregulation, and apoptotic signaling are key central mechanisms underlying SNHL, whereas endogenous defense systems involving SOD, CAT, GPx, and the Nrf2/HO‐1 pathway provide only limited protection [23]. ROS generated during reperfusion and inflammatory signaling from the stria vascularis and spiral ligament contribute to cochlear hypoxia and apoptotic degeneration. In contrast, microcirculatory impairment exacerbates injury by disrupting endocochlear potential and endolymph homeostasis [3, 23]. Accordingly, exogenous antioxidants with redox‐balancing and otoprotective properties represent rational therapeutic targets for countering ROS‐mediated cochlear damage.

Assessment of diabetic auditory neuropathy using ABR is an important tool because it can detect neural dysfunction before overt hearing loss becomes clinically apparent [24]. In a study using middle‐aged CBA/CaJ mice, T1DM induced by STZ and T2DM induced by high‐fat diet both resulted in elevated ABR thresholds at 6 months, with earlier and more pronounced changes observed in the T2DM model [25]. The absence of detailed ABR‐based auditory assessments in the STZ–NA model of T2DM underscores the novelty of our study. Furthermore, the ABR protocol used here provided robust and reproducible auditory analysis, with representative Wave II responses at 16 kHz illustrated to demonstrate threshold determination and waveform morphology. In the present study, ABR findings reflect functional auditory alterations associated with T2DM‐related oxidative stress rather than a definitive diagnostic classification. While ABR provides valuable information on auditory pathway function, a comprehensive evaluation of auditory neuropathy typically requires complementary assessments, including otoacoustic emissions to confirm outer hair cell integrity.

Diabetic neuropathy is a common microvascular complication that arises from chronic hyperglycemia, which increases oxidative stress in part through the glycosylation and inactivation of endogenous antioxidant enzymes. Polyphenols exert neuroprotective effects by reducing oxidative injury and preserving neurovascular functions [10]. Notably, elevated glucose levels can cause hearing loss even during the prediabetic stage, with ROS playing a central pathogenic role [26]. Therefore, effective glycemic control is critical for preserving auditory functions. The inner ear is particularly vulnerable to ischemia and oxidative damage. Agents that enhance cochlear blood flow, strengthen antioxidant defense, and suppress inflammatory signaling have been reported to exert otoprotective effects [3]. In this context, flavonoids may help maintain redox homeostasis, limit inflammation and apoptosis, and mitigate mitochondrial dysfunction, thereby potentially supporting cochlear function. Given their low toxicity and favorable safety profiles, natural antidiabetic compounds are promising therapeutic alternatives. Recent studies have demonstrated that the phenolic compounds eugenol and quercetin exhibit antioxidant and antidiabetic properties, particularly in the STZ–NA‐induced experimental T2DM model [18]. In our study, the marked increase in serum MDA levels in the T2DM group clearly indicated significant oxidative stress, whereas the reduction in SOD indicated compromised antioxidant capacity. Treatment with eugenol (20 mg/kg) or quercetin (50 mg/kg) partially restored redox balance by decreasing MDA and increasing SOD activity, supporting the strong antioxidant efficacy of both compounds and their protective potential against diabetes‐associated oxidative stress. In parallel, serum GHbA1c levels were significantly higher in the T2DM group than in controls (*p* < 0.01). Eugenol, but not quercetin, significantly reduced GHbA1c levels relative to untreated T2DM (*p* < 0.05), indicating that the antioxidant effects observed—particularly with quercetin—may occur, at least in part, independently of glycemic control. Nevertheless, further studies incorporating long‐term glycemic markers are warranted to confirm this interpretation (Figure 7a–c).

**Figure 3:**
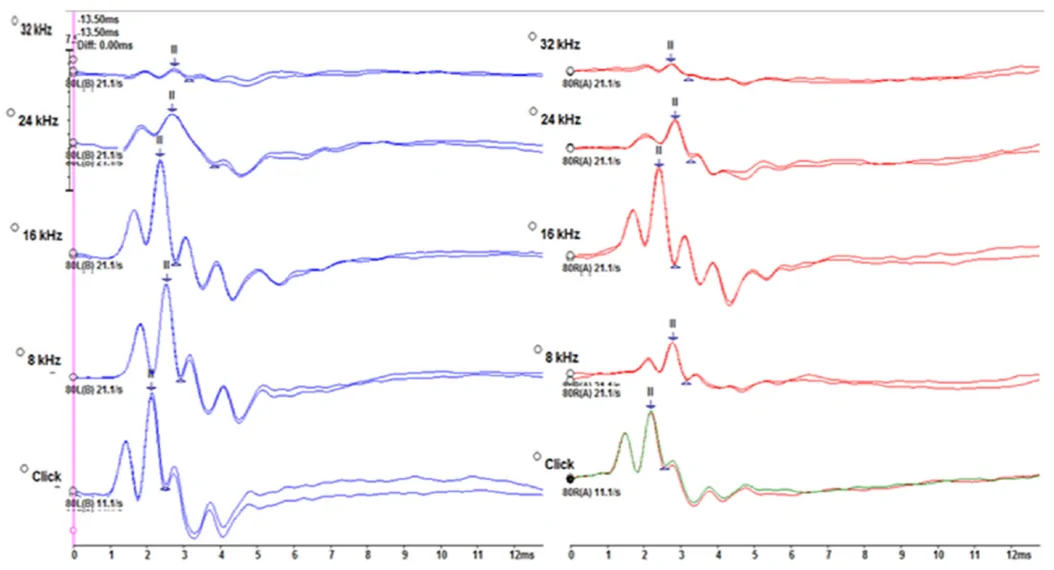
Representative ABR waveforms elicited by click and tone‐burst stimuli (8, 16, 24, and 32 kHz) at 80 dB SPL in the right (red) and left (blue) ears of a control rat, showing prominent Wave II responses.

The hearing range in Sprague–Dawley rats spans approximately 250 Hz–80 kHz [27]. Borg [28] reported that the maximal auditory sensitivity occurs between 12 and 24 kHz, within which 16 kHz provides the lowest hearing thresholds and the clearest waveform morphology in ABR analyses [29]. This frequency range is considered relatively preserved due to more balanced cochlear blood flow and stronger antioxidant defenses, in contrast to the higher metabolic demand and oxidative vulnerability of the basal cochlear region at 24 kHz and the greater degenerative susceptibility of the apical region at 8 kHz. In this study, 16 kHz was identified as the most informative frequency for both threshold determination and waveform morphology (Figures 2 and 3). These findings suggest that 16 kHz is an advantageous target frequency for early detection of diabetes‐related auditory dysfunction, monitoring of disease progression, and evaluation of treatment efficacy. Alvarado et al. [19] reported that Wave II is the most prominent ABR component in healthy Wistar rats and that responses at 32 kHz are typically observed as single peaks. Similarly, this study confirmed that Wave II was the dominant response in Sprague–Dawley rats (Figure 3). Previous studies have also shown that ABR thresholds elicited by click stimuli in diabetic models are comparable to those obtained with 8 kHz tone bursts [30]. In agreement with these findings, the Wave II morphologies obtained using click and 8 kHz stimuli at 80 dB SPL were similar in this study (Figure 3).

**Figure 4:**
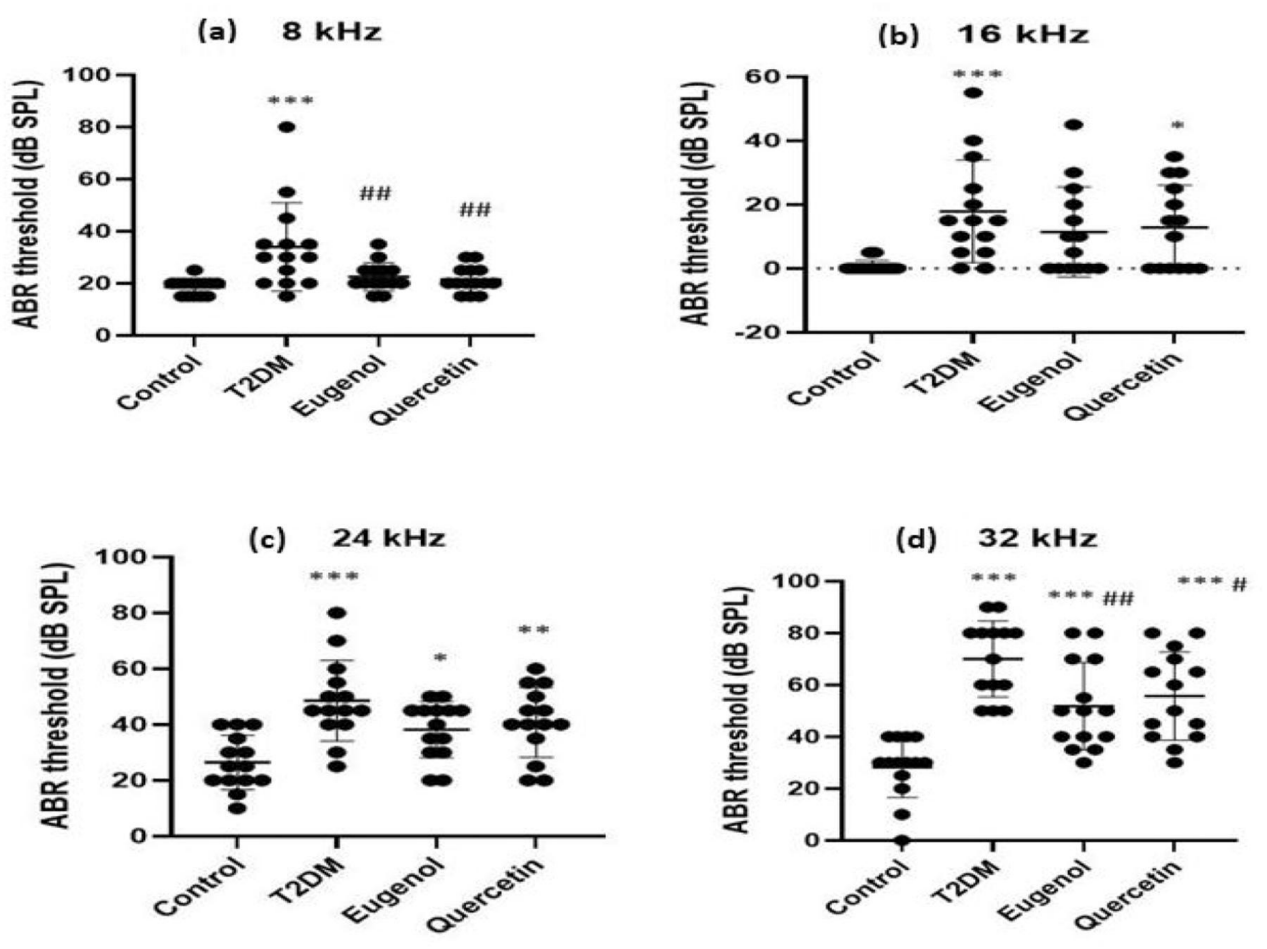
ABR threshold values at different frequencies: (a) 8 kHz, (b) 16 kHz, (c) 24 kHz, and (d) 32 kHz in the control, T2DM, eugenol, and quercetin groups. Values are shown as individual data points (*n* = 14 ears per group; left and right). *p < 0.05, **p < 0.01, ***p < 0.001 versus control group; ^#^ *p* < 0.05, ^##^ *p* < 0.01 versus T2DM group. Statistical analysis was performed using the Kruskal–Wallis test followed by Dunn's multiple comparisons test.

Wave II, the most prominent component of the ABR waveform in rats, is thought to originate from the cochlear nucleus complex and, due to its high neural firing activity, has been suggested to correspond functionally to Wave III in humans [11]. Alvarado et al. [19] reported that Wave II was the most prominent in Wistar rats. In contrast, Wave III is often undetectable at low frequencies and typically appears as small complexes combined with Wave IV. These complexes were reported to be common at low‐to‐mid frequencies but rare at high frequencies. Similarly, Jafarzadeh and Pourbakht [31] demonstrated in an ABR study using click stimuli in rats that Wave II is the most reliable reference across frequencies. In contrast, Waves III and V exhibit low amplitudes and are frequently observed in merged forms. Accordingly, the characteristic ABR morphology in rats can be described as a dominant Wave II originating from the cochlear nucleus, a small‐amplitude Wave III that is often contiguous with Wave IV, prominent IV–V complexes at 8–16 kHz that tend to diminish at 24–32 kHz, and progressive loss of neural synchronization with increasing stimulus frequency. In this study (Figures 2 and 3), the consistent dominance of Wave II across all tested frequencies, the presence of low‐amplitude Wave III adjacent to Wave IV, and the infrequent observation of complex waveforms at higher frequencies are consistent with prior reports and reflect frequency‐dependent synchronization dynamics in rat auditory pathways. Collectively, these findings support the methodological validity of the ABR wave morphology used for interpretation under diabetic and treatment conditions.

Previous studies have demonstrated diabetes‐related elevations in ABR thresholds across experimental models. In mice with STZ‐induced T1DM and genetically induced T2DM, red Korean ginseng suppressed threshold elevation only in the T2DM model after 8 weeks, with no improvement observed in T1DM, and no significant changes in wave latencies [32]. Similarly, in an STZ‐induced C57BL/6 mouse model, a significant increase in ABR thresholds at 4 kHz was reported after 4 weeks [33]. Consistent with these findings, this study demonstrated significantly elevated hearing thresholds at 8, 16, 24, and 32 kHz on Day 49 in the STZ–NA‐induced T2DM model compared with the control group (Figure 4a–d and Table 1). When analyzed by frequency, quercetin showed a significant functional protective effect at 16 kHz (*p* < 0.05), both eugenol and quercetin were functionally protective at 24 kHz (*p* < 0.05), and both agents showed significant functional effects at 32 kHz (*p* < 0.01 and *p* < 0.05, respectively). Across all groups, the lowest thresholds were observed at 16 kHz, whereas the highest thresholds occurred at 32 kHz. The observed predominance of threshold elevation at higher frequencies (24–32 kHz) is consistent with previous experimental studies demonstrating preferential high‐frequency cochlear involvement in diabetes, particularly at 32 kHz [9, 34], as also summarized in recent systematic reviews [2]. This pattern is likely related to the increased metabolic demand and microvascular susceptibility of the basal turn of the cochlea, which renders high‐frequency regions more vulnerable to oxidative stress–mediated damage [35]. The increase in hearing thresholds across all test frequencies in the T2DM group indicates that even lower frequencies, such as 8 kHz, are susceptible to hyperglycemia‐induced auditory dysfunction. At 32 kHz, both eugenol and quercetin attenuated the diabetes‐associated elevation in ABR thresholds, with eugenol producing a more pronounced improvement than quercetin. The more pronounced threshold increases at 24 and 32 kHz further suggest that ABR measurements may be particularly sensitive for detecting high‐frequency auditory impairment in experimental rat models of diabetes.

**Figure 5:**
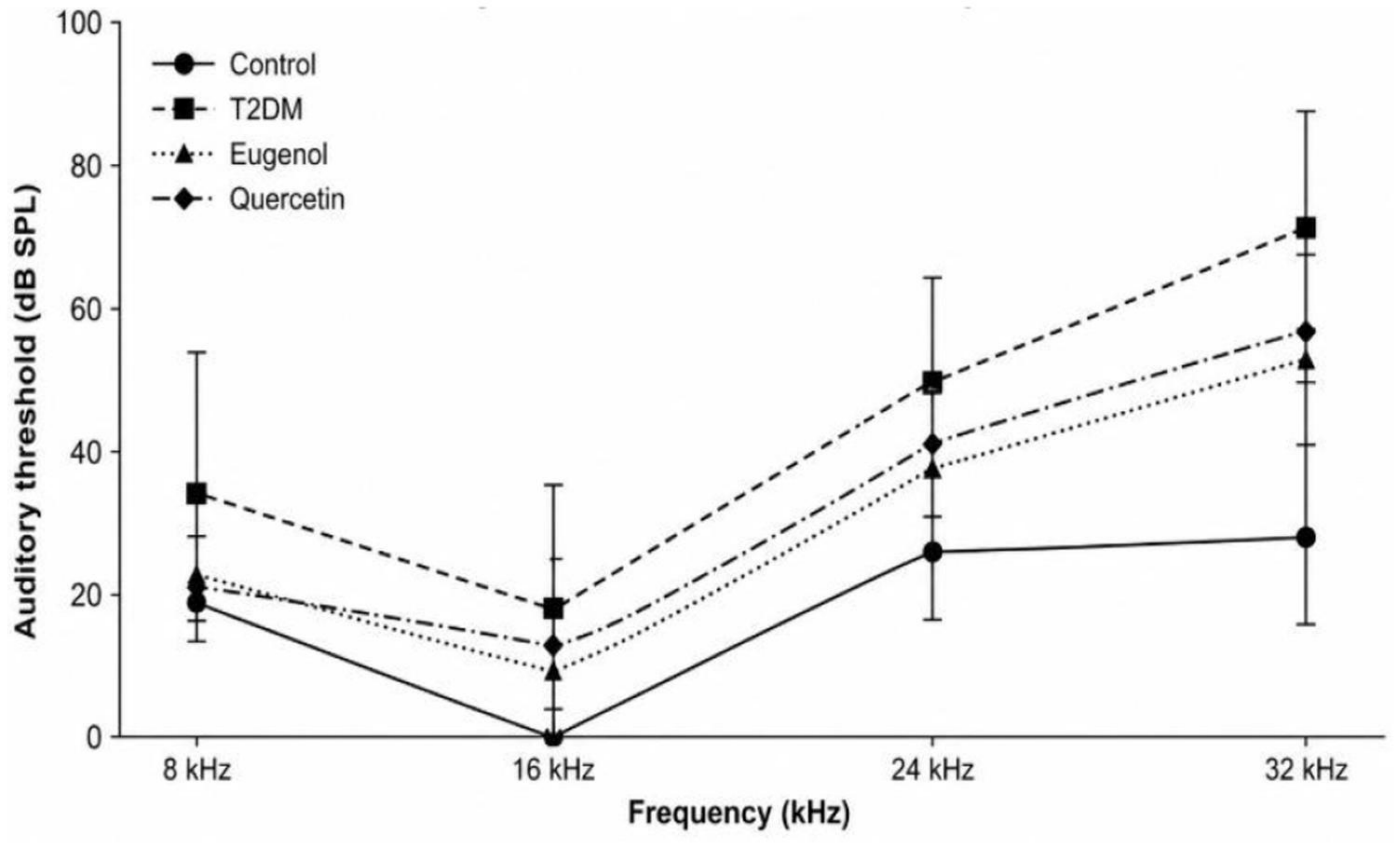
Frequency‐specific auditory threshold values (dB SPL) at 8, 16, 24, and 32 kHz in control, T2DM, eugenol‐treated, and quercetin‐treated groups. Statistical comparisons were performed as described in Table 1.

Inflammation and excessive ROS production are central to the pathogenesis of DM, positioning naturally occurring phenolic antioxidants as promising therapeutic candidates. Quercetin, a flavonoid with potent antioxidant activity, has attracted considerable interest for preventing diabetes‐related neurological complications due to its ability to enhance endogenous antioxidant defenses, attenuate oxidative damage, regulate metabolic processes, and modulate intracellular signaling pathways [22]. In experimental diabetic models, quercetin has been reported to preserve neuronal and glial cell density, reduce cognitive impairment, and exert neuroprotective effects by suppressing oxidative stress [36, 37]. Eugenol, a phenolic compound of the phenylpropanoid class, exhibits strong antioxidant, anti‐inflammatory, and antimicrobial properties. It effectively suppresses lipid peroxidation and is considered safe by the US Food and Drug Administration [38]. In diabetic models, eugenol has been shown to reduce inflammation and oxidative stress [15], enhance insulin sensitivity by activating the AMPK/GLUT4 signaling pathway, and restore the balance between GSH and MDA [16]. Eugenol has also demonstrated otoprotective effects by reversing MDA elevation and GPx‐SOD depletion in a cisplatin‐induced ototoxicity model [38]. Hearing loss is prevalent among patients with T2DM and predominantly affects high‐frequency hearing, with diabetic neuropathic mechanisms proposed as a significant contributing factor [6]. Consistent with this concept, improvements in ABR thresholds (Table 1 and Figure 5) indicate that both quercetin and eugenol partially attenuate diabetes‐related functional auditory impairment associated with oxidative stress, particularly at higher frequencies (24–32 kHz). These results support the notion that metabolically demanding cochlear regions are more susceptible to hyperglycemia‐induced oxidative stress and microvascular impairment. Moreover, they demonstrate that high‐frequency ABR measurements (24–32 kHz) provide a sensitive approach for detecting diabetes‐related auditory dysfunction and for evaluating the efficacy of antioxidant interventions.

**Figure 6:**
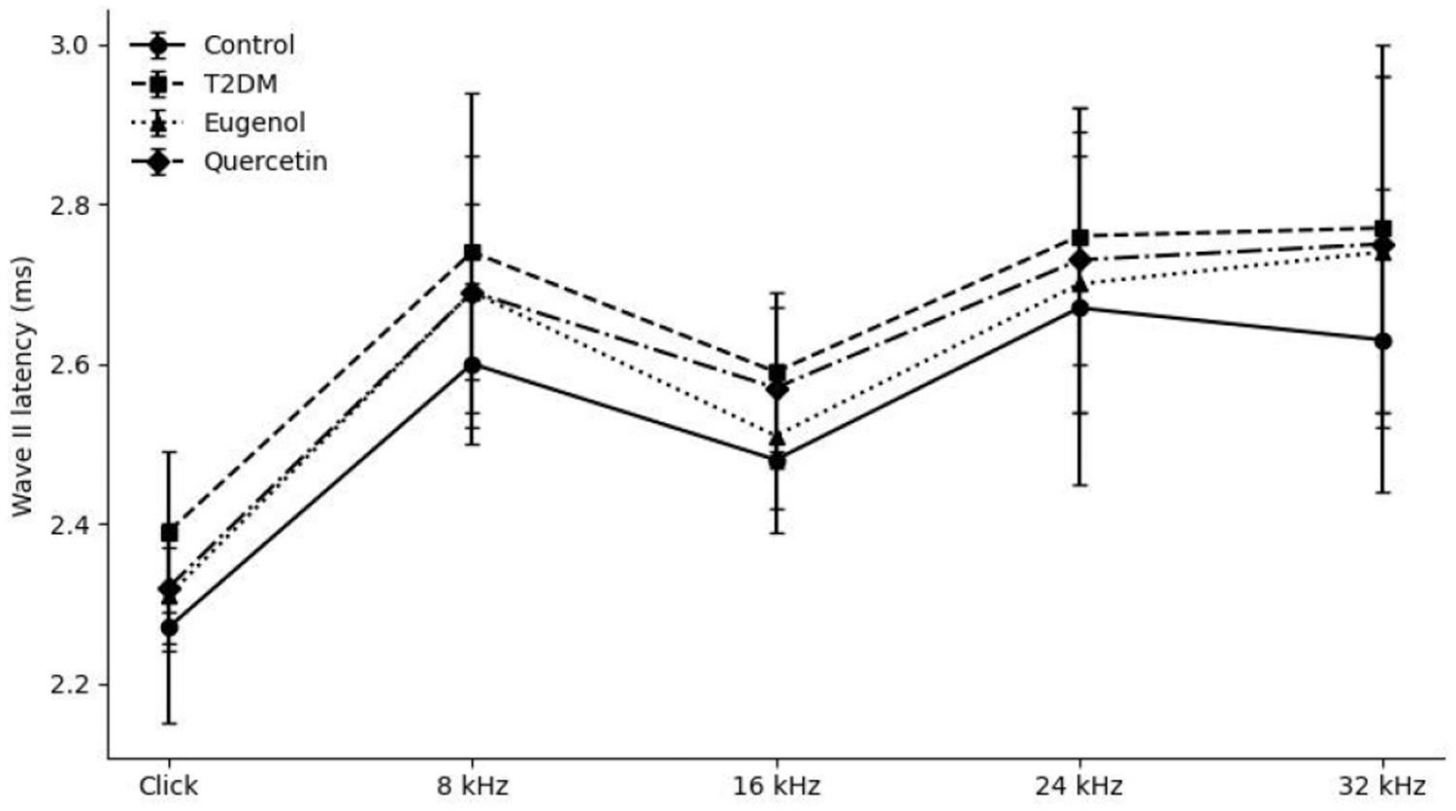
Wave II latency values (ms) across stimulus types (click and tone burst at 8, 16, 24, and 32 kHz at 80 dB SPL) in control, T2DM, eugenol‐treated, and quercetin‐treated groups. Statistical comparisons were performed as described in Table 2.

In the T2DM group, prolonged Wave II latencies across all frequencies compared with controls (Table 2) indicate slowed neural transmission along the auditory pathway. Statistically significant prolongations at 16 kHz (*p* = 0.018) and click stimuli (*p* = 0.017) may represent early electrophysiological indicators of diabetes‐related auditory dysfunction. Although differences at 8, 24, and 32 kHz did not reach statistical significance, a consistent trend toward increased Wave II latency was observed. The delayed and attenuated Wave II responses in the T2DM rats (Figures 2, 6 and Table 2) are consistent with impaired neural conduction attributable to oxidative stress and microvascular dysfunction. The administration of eugenol or quercetin 21 days after diabetes induction resulted in significant improvements in hearing thresholds (Table 1 and Figure 5). These findings suggest that diabetic auditory dysfunction is characterized by frequency‐dependent threshold elevation and prolonged Wave II latency, both associated with metabolic stress, and that antioxidant treatment can partially modulate these alterations. Notably, improvements at 24–32 kHz support the concept that early antioxidant intervention may attenuate the progression of diabetes‐related high‐frequency hearing impairment. Preservation of Wave II morphology and intermediate latency values between the control and untreated T2DM groups in the treatment cohorts suggests partial functional improvement in auditory neural conduction. Whereas elevated high‐frequency thresholds may be consistent with outer hair cell dysfunction, prolonged Wave II latencies may reflect impaired synaptic synchronization or degeneration of the spiral ganglion; however, these cellular and structural mechanisms cannot be confirmed by ABR alone. Collectively, these findings suggest that exogenous antioxidant therapy may improve auditory sensitivity, neural synchronization, and conduction velocity.

**Figure 7:**
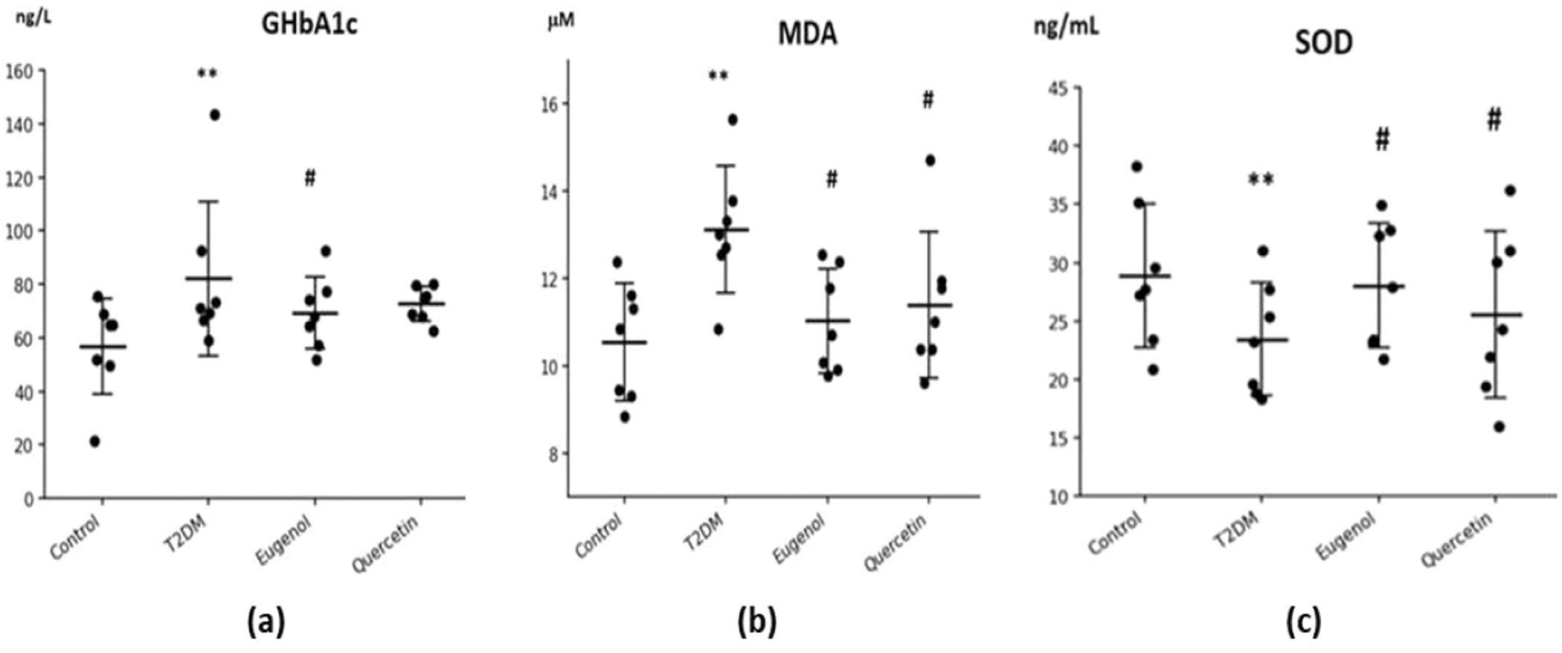
Serum biomarker levels in the control, T2DM, eugenol‐treated, and quercetin‐treated groups: (a) GHbA1c, (b) MDA, and (c) SOD. Values are expressed as mean ± SD (*n* = 7). **p < 0.01 versus control group; ^#^ *p* < 0.05 versus T2DM group. Statistical analyses were performed using the Kruskal–Wallis test followed by Dunn's multiple comparisons test for GHbA1c, and one‐way ANOVA followed by Tukey's post hoc test for MDA and SOD.

Overall, our findings demonstrate that diabetes‐related high‐frequency auditory dysfunction in the STZ–NA–induced T2DM model is closely associated with oxidative stress, independent of glycemic control, as evidenced by MDA–SOD correlations and regression analyses. Eugenol and quercetin conferred partial functional protection of auditory pathways, as reflected by improved high‐frequency ABR thresholds and Wave II latencies. A proposed mechanistic interpretation of the relationships among hyperglycemia, oxidative stress, auditory dysfunction, and the antioxidant‐mediated protective effects of quercetin and eugenol is presented in Figure 8.

**Figure 8:**
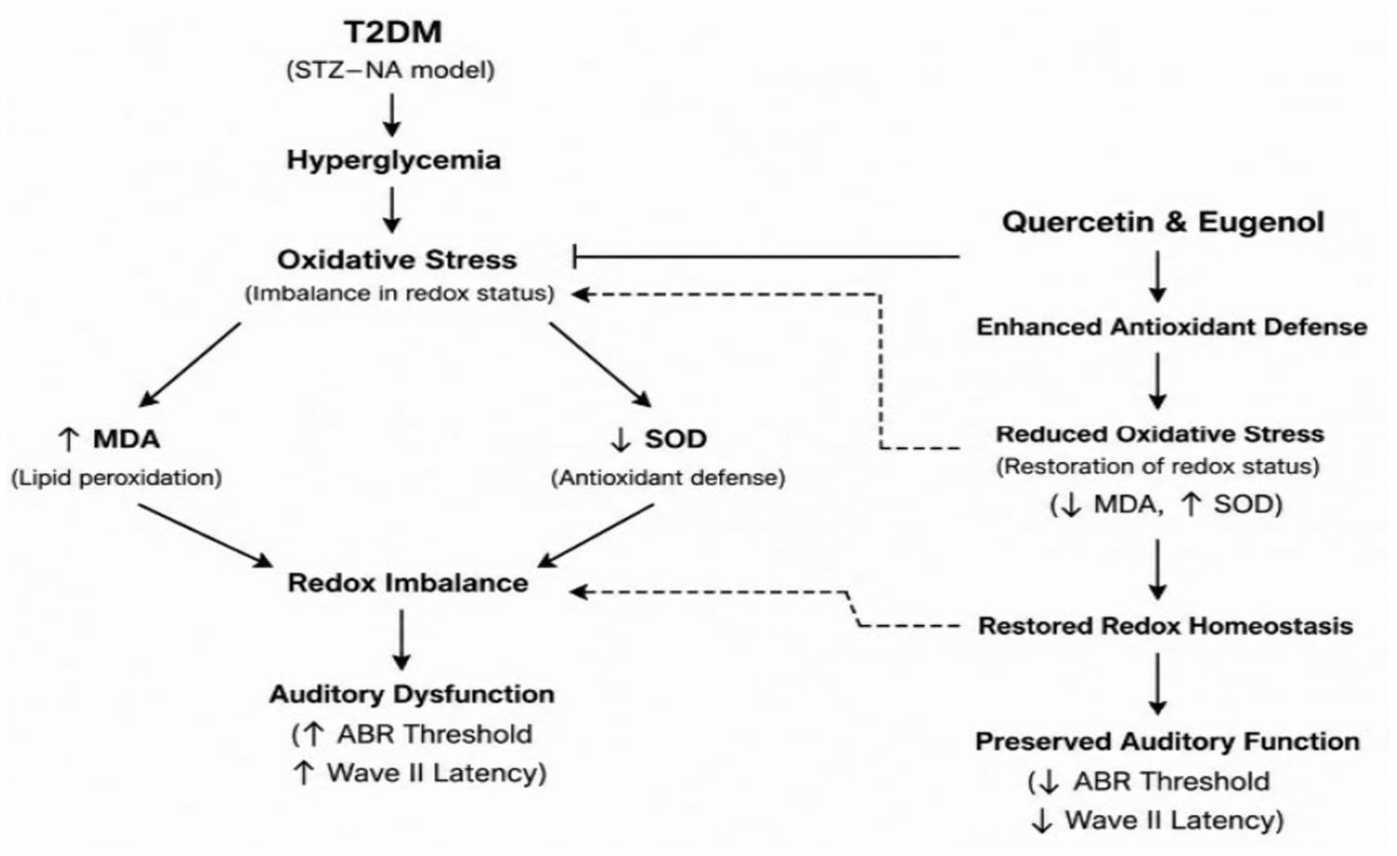
Mechanistic model of oxidative stress–mediated auditory dysfunction and quercetin‐ and eugenol‐mediated functional auditory preservation in experimental T2DM.

These findings support the utility of 24–32 kHz ABR measurements as sensitive experimental markers of diabetes‐associated auditory dysfunction while underscoring the need for further mechanistic and translational investigation. A limitation of the present study is the absence of complementary morphological and immunohistochemical evaluation of cochlear hair cells, as well as neural and synaptic components. Accordingly, although improvements in ABR thresholds and Wave II latencies suggest partial preservation of auditory pathway function, these findings should be interpreted as functional electrophysiological rather than direct structural evidence, and future studies integrating high‐resolution cellular analyses would further strengthen mechanistic understanding.

In light of the reported susceptibility to high‐frequency hearing impairment among patients with T2DM, frequency‐specific ABR assessment may facilitate earlier recognition of subclinical auditory involvement and serve as a complementary modality to routine pure‐tone audiometry in the longitudinal management of diabetes; nevertheless, its integration into standard clinical practice warrants validation in well‐designed prospective human studies.

## 5 Conclusion

In conclusion, diabetes‐related auditory dysfunction in the STZ–NA‐induced T2DM model was confirmed by increased ABR thresholds and prolonged Wave II latencies, underscoring the central role of oxidative stress in diabetes‐associated auditory impairment. Eugenol and quercetin partially attenuated these electrophysiological alterations, particularly at higher frequencies, consistent with their antioxidant properties. These findings support the utility of ABR testing for the early detection of hyperglycemia‐related auditory dysfunction and suggest that antioxidant‐based interventions may modulate disease progression. The present experimental findings suggest that diabetes‐related oxidative stress may increase vulnerability to high‐frequency hearing impairment, supporting attentive auditory follow‐up in patients with T2DM, with rehabilitation considered when clinically appropriate. Given that the present evidence is derived from an experimental animal model and considering the low toxicity and favorable safety profiles of both compounds, eugenol and quercetin should be regarded as promising candidates for further mechanistic and translational investigation rather than for direct clinical application, pending confirmation in well‐designed human studies.

## Author Contributions

**Aycan Baş:** conceptualization, methodology, formal analysis, data curation, writing – original draft, supervision, project administration, funding acquisition. **Cem Doğan:** investigation, formal analysis, writing – review and editing. **Işıl Albeni̇z:** conceptualization, supervision, project administration, writing – review and editing. All authors approved the final version of the manuscript; agree to be accountable for all aspects of the work in ensuring that questions related to the accuracy or integrity of any part of the work are appropriately investigated and resolved; and all persons designated as authors qualify for authorship, and all those who qualify for authorship are listed.

## Ethics Statement

All animal care and handling procedures were approved by the Local Ethics Committee for Animal Experiments of Istanbul University (Approval No. 2016/26) and conducted in accordance with national regulations for the care and use of laboratory animals. The procedures were consistent with the principles of the European Directive 2010/63/EU and were reported in accordance with the ARRIVE guidelines.

## Conflicts of Interest

The authors declare no conflicts of interest.

## Supporting information


Supporting File 1

Supporting File 2

## Data Availability

The data that support the findings of this study are available from the corresponding author upon reasonable request.
