# Supplementary material for: Protective Effects of Quercetin and Eugenol on Auditory Dysfunction Associated With Oxidative Stress in a Type 2 Diabetic Rat Model
Source: J Biochem Mol Toxicol. 2026 Nov 15;40(11):e71118. doi: 10.1002/jbt.71118 (PMC13770424; doi:10.1002/jbt.71118)
Supplement: Supplementary file 1 [file JBT-40-e71118-s001.docx]

**Supplementary Figures S1–S4.** Frequency-specific representative ABR waveforms illustrating threshold determination across experimental groups (control, diabetic, eugenol, and quercetin).

**Figure S1.** Representative ABR waveforms at 8 kHz tone-burst stimuli used for threshold determination in the control, diabetic, eugenol, and quercetin groups (right ear).

**Figure S2.** Representative ABR waveforms at 16 kHz tone-burst stimuli used for threshold determination in the control, diabetic, eugenol, and quercetin groups (left ear).

**Figure S3.** Representative ABR waveforms at 24 kHz tone-burst stimuli used for threshold determination in the control, diabetic, eugenol, and quercetin groups (right ear).

**Figure S4.** Representative ABR waveforms at 32 kHz tone-burst stimuli used for threshold determination in the control, diabetic, eugenol, and quercetin groups (left ear).
