# Supplementary material for: Protective Effects of Quercetin and Eugenol on Auditory Dysfunction Associated With Oxidative Stress in a Type 2 Diabetic Rat Model
Source: J Biochem Mol Toxicol. 2026 Nov 15;40(11):e71118. doi: 10.1002/jbt.71118 (PMC13770424; doi:10.1002/jbt.71118)
Supplement: Supplementary file 2 [file JBT-40-e71118-s002.docx]

**Table 1.** Body weight measurements at baseline, Day 21, and Day 49 in the experimental T2DM model

| **Time point** | **Control** | **T2DM** | **Eugenol** | **Quercetin** |
| --- | --- | --- | --- | --- |
| **Baseline** | 261.57 ± 2.22 | 266.57 ± 30.44 | 261.85 ± 34.28 | 266.00 ± 38.29 |
| **Pre-treatment (Day 21)** | 291.42 ± 4.18ᵃ | 275.85 ± 52.97 | 269.00 ± 46.69ᵇ | 267.71 ± 32.10ᵇ |
| **End-point (Day 49)** | 314.42 ± 3.82ᵃ | 268.42 ± 30.82ᵇ | 276.71 ± 32.43ᵇ | 279.57 ± 22.88ᵇ |

# Values are expressed as mean ± SD (n = 7). Different superscript letters (a, b) within the same row indicate significant differences between groups (p < 0.05). Group comparisons were performed using the Kruskal–Wallis test followed by the Mann–Whitney U test.

**Table 2.** Fasting blood glucose levels at baseline, day 21, and end of the experiment in the experimental T2DM model

| **Time point** | **Control** | **T2DM** | **Eugenol** | **Quercetin** |
| --- | --- | --- | --- | --- |
| **Baseline** | 111.71 ± 2.22ᵃ | 380.85 ± 30.44ᵇ | 448.28 ± 34.28ᵇ | 371.00 ± 38.29ᵇ |
| **Pre-treatment (Day 21)** | 108.57 ± 4.19ᵃ | 324.42 ± 52.97ᵇ | 344.71 ± 46.69ᵇ | 344.00 ± 32.10ᵇ |
| **End-point (Day 49)** | 112.14 ± 3.82ᵃ | 403.28 ± 30.82ᶜ | 273.42 ± 32.43ᵇ | 213.42 ± 22.88ᵇ |

Values are expressed as mean ± SD (n = 7). Different superscript letters (a–c) within the same row indicate significant differences between groups (p < 0.05). Group comparisons were performed using the Kruskal–Wallis test followed by the Mann–Whitney U test.
